# Exploring an objective measure of overactivity in children with rare genetic syndromes

**DOI:** 10.1186/s11689-024-09535-y

**Published:** 2024-04-18

**Authors:** Rory O’Sullivan, Stacey Bissell, Georgie Agar, Jayne Spiller, Andrew Surtees, Mary Heald, Emma Clarkson, Aamina Khan, Christopher Oliver, Andrew P. Bagshaw, Caroline Richards

**Affiliations:** 1 School of Psychology, University of Birmingham, Birmingham, UK; 2 Cerebra Network for Neurodevelopmental Disorders, University of Birmingham, Birmingham, UK; 3 School of Life & Health Sciences, Aston University, Birmingham, UK; 4 School of Psychology and Vision Sciences, University of Leicester, Leicester, UK; 5 Blackpool Teaching Hospitals NHS Foundation Trust, Blackpool, Lancashire UK; 6 The Huntercombe Group, Worcestershire, UK; 7 Centre for Human Brain Health, University of Birmingham, Birmingham, UK

**Keywords:** Actigraphy, Overactivity, Hyperactivity, Rare genetic syndromes, Children, Objective, Questionnaire, Smith-Magenis syndrome, Angelman syndrome, Tuberous sclerosis complex

## Abstract

**Background:**

Overactivity is prevalent in several rare genetic neurodevelopmental syndromes, including Smith-Magenis syndrome, Angelman syndrome, and tuberous sclerosis complex, although has been predominantly assessed using questionnaire techniques. Threats to the precision and validity of questionnaire data may undermine existing insights into this behaviour. Previous research indicates objective measures, namely actigraphy, can effectively differentiate non-overactive children from those with attention-deficit hyperactivity disorder. This study is the first to examine the sensitivity of actigraphy to overactivity across rare genetic syndromes associated with intellectual disability, through comparisons with typically-developing peers and questionnaire overactivity estimates.

**Methods:**

A secondary analysis of actigraphy data and overactivity estimates from The Activity Questionnaire (TAQ) was conducted for children aged 4-15 years with Smith-Magenis syndrome (*N*=20), Angelman syndrome (*N*=26), tuberous sclerosis complex (*N*=16), and typically-developing children (*N*=61). Actigraphy data were summarized using the M10 non-parametric circadian rhythm variable, and 24-hour activity profiles were modelled via functional linear modelling. Associations between actigraphy data and TAQ overactivity estimates were explored. Differences in actigraphy-defined activity were also examined between syndrome and typically-developing groups, and between children with high and low TAQ overactivity scores within syndromes.

**Results:**

M10 and TAQ overactivity scores were strongly positively correlated for children with Angelman syndrome and Smith-Magenis syndrome. M10 did not substantially differ between the syndrome and typically-developing groups. Higher early morning activity and lower evening activity was observed across all syndrome groups relative to typically-developing peers. High and low TAQ group comparisons revealed syndrome-specific profiles of overactivity, persisting throughout the day in Angelman syndrome, occurring during the early morning and early afternoon in Smith-Magenis syndrome, and manifesting briefly in the evening in tuberous sclerosis complex.

**Discussion:**

These findings provide some support for the sensitivity of actigraphy to overactivity in children with rare genetic syndromes, and offer syndrome-specific temporal descriptions of overactivity. The findings advance existing descriptions of overactivity, provided by questionnaire techniques, in children with rare genetic syndromes and have implications for the measurement of overactivity. Future studies should examine the impact of syndrome-related characteristics on actigraphy-defined activity and overactivity estimates from actigraphy and questionnaire techniques.

**Supplementary Information:**

The online version contains supplementary material available at 10.1186/s11689-024-09535-y.

## Introduction

Overactivity, also referred to as hyperactivity, is primarily characterised by excessive gross motor activity and commonly described via hypermotoric behaviours such as restlessness, an inability to sit still, repeated movements, or ‘acting as if being driven by a motor’ [1, 2]. Overactivity is characteristic of several neurodevelopmental conditions, in particular hyperactive-impulsive and combined presentations of attention-deficit hyperactivity disorder (ADHD), where overactive behaviours are essential for diagnosis [1, 2]. Several rare genetic syndromes associated with intellectual disability evidence heightened rates of overactivity, amongst these syndromes are Smith-Magenis syndrome (SMS, 74.2-94.0% [3, 4], tuberous sclerosis complex (TSC, 54.8-56.0% [5, 6]), and Angelman syndrome (AS, 50.0-94.0% [7]). The clinical significance of overactivity in neurodevelopmental conditions, broadly, and rare genetic syndromes, more specifically, is highlighted by associations with greater caregiving challenges and stress [8, 9], persistent self-injury [10–12] and aggressive behaviour [12–14], as well as sleep difficulties and disorders [15–20]. Recent evidence also indicates that overactivity is associated with lower self-help abilities within specific rare genetic syndrome populations [21, 22]. The relevance of overactivity to caregiving experiences, persistent challenging behaviours and poor sleep highlights the need to further investigate this behaviour in rare genetic syndromes, using refined and robust methods of assessment.

Several subjective and objective techniques are available to quantify overactivity in children with neurodevelopmental conditions. Subjective measures predominantly include teacher- and caregiver-completed questionnaires and clinical interviews [23]; objective measures are more diverse, including infrared motion analysis [24], video compression algorithms [25] and actigraphy [26]. Within rare genetic syndrome research, overactivity is almost universally quantified using caregiver-completed questionnaires (e.g. [27, 28]). Examples include standardized questionnaires such as The Activity Questionnaire [29], Aberrant Behavior Checklist [30] and Vanderbilt ADHD Rating Scales [31] as well as study-specific questionnaires (e.g. [7]). Questionnaires typically list behaviours associated with overactivity and caregivers rate the presence, severity or frequency of these over a specified time period, often via Likert scales. Ratings are summarized into total or subscale scores, providing single global estimates of children’s overactivity.

Questionnaires produce valuable data across research and clinical settings as these draw on caregivers’ and teachers’ experience with children, have low technical and financial demands, and are sensitive to specific qualitative characteristics of overactivity (e.g. discomfort remaining still) [32]. However, the limitations of questionnaires raises concerns regarding the sole use of this technique to evaluate overactivity throughout rare genetic syndrome research, and highlights the need for additional complementary measures. Firstly, global estimates of overactivity do not capture variation in children’s overactivity across time [33, 34], highlighting the need for measures sensitive to this temporal variation. Secondly, recall and informant-report biases may impact questionnaire overactivity estimates [35–38], necessitating measures of overactivity resistant to these biases. Finally, overactivity estimates from questionnaires and objective techniques, such as actigraphy and motion tracking systems, demonstrate relatively poor concordance in overactive [39, 40] and non-overactive children [41]. Threats to the convergent validity of questionnaires and objective activity measures raises concerns, as objective techniques directly measure motor activity, heightened levels of which inherently characterise overactivity [1, 2]. These limitations highlight the need to introduce alternative, complementary measures of overactivity to rare genetic syndrome research, alongside questionnaires, to obtain a more robust and comprehensive understanding of overactivity in these populations.

Research with other overactive populations, particularly ADHD, supports the sensitivity of actigraphy to overactivity [26, 42]. Typically used to estimate sleep parameters and physical activity profiles, actigraphic devices are worn on participants’ wrists, ankles or hips, and contain accelerometers that measure acceleration generated by body movements. Depending on specific hardware and processing algorithms, actigraphy can quantify the frequency, duration and/or intensity of movements [43]. Meta-analyses that have pooled these metrics have revealed elevated levels of activity in children with ADHD compared to typically-developing (TD) peers [26, 42]. Previous ADHD studies have also summarized actigraphy data utilizing the M10 non-parametric circadian rhythm variable which describes the magnitude of activity during the most active 10 hours of the day [44]. These studies have revealed greater M10 activity levels in individuals with ADHD compared to TD controls [45–49]. This highlights the sensitivity of actigraphy to children’s overactivity, indicating that, in addition to questionnaires, actigraphy may facilitate robust assessment of overactivity in children with rare genetic syndromes.

Actigraphy offers several advantages for evaluating overactivity. Firstly, as an objective technique, actigraphy is resistant to recall and informant-report biases, and quantifies activity consistently across different environments, promoting the validity and reliability of overactivity estimates. Secondly, actigraphy is well-tolerated by children with rare genetic syndromes (e.g. [50–53]) and can be continuously worn over sustained periods, typically up to one week. Therefore, actigraphy can measure intra- and inter-day variation in children’s activity, increasing the representativeness of overactivity estimates, and capturing children’s 24-hour activity profiles from which the timing, frequency and duration of heightened activity can be precisely delineated. Consequently, actigraphy can overcome biases associated with questionnaires, and provide complementary description of children’s activity that can advance existing understanding of overactivity in rare genetic syndromes.

Although previous studies of rare genetic syndromes have measured children’s physical activity using actigraphy, none have rigorously examined the sensitivity of actigraphy to overactivity [54–58]. No published study has directly addressed the convergent validity of actigraphy-defined activity with established overactivity questionnaires used in rare syndrome research. Existing evidence is limited to correlations between actigraphy-defined activity and ADHD subscale scores, confounded by the inclusion of overactive, impulsive and inattentive behaviours [54]. Additionally, this study only collected actigraphy data during short laboratory sessions, limiting the likelihood of capturing children’s naturalistic overactive behaviours. Although studies have compared actigraphy-measured activity between children with genetic syndromes and TD peers, these often include syndromes not associated with overactivity, such as Down syndrome [59]. Many studies also conduct comparisons with TD siblings, introducing bias as siblings’ activity levels may be influenced by their overactive siblings (i.e. carryover effects, see [60]). Furthermore, only one study has compared 24-hour activity profiles between a genetic syndrome sample, specifically Sanfilippo syndrome, and a TD control group [57]. However, the findings were limited as activity data were summarized in 6-hour bins, obscuring the exact timing and duration of heightened physical activity, and likely overlooking short periods of heightened activity. Minute-by-minute comparisons of activity across the 24-hour cycle are required to precisely examine the naturalistic temporal profile of overactivity. Therefore, to determine the sensitivity of actigraphy to overactivity in children with rare genetic syndromes, research should: (i) capture naturalistic overactive behaviours via multi-day actigraphy assessments in children’s natural environments; (ii) explore differences in actigraphy-measured activity profiles between syndrome groups associated with overactivity and an independent sample of TD peers; (iii), establish the convergence of actigraphy data and questionnaire overactivity estimates in syndromes associated with overactivity; and (iv), infer the timing, frequency, and duration of overactivity within 24-hour syndrome-specific activity profiles.

The current study will address the limitations of previous work to delineate the sensitivity of actigraphy to overactivity in children with rare genetic syndromes. The research aims are as follows:

1. Explore the convergence of actigraphy-defined activity and questionnaire overactivity estimates, to further understand the properties of these measures.
2. Identify differences in actigraphy activity data, summarised via the M10 statistic and 24-hour activity profiles, between children with rare genetic syndromes associated with overactivity and TD controls.

## Methods

### Study design and participants

This study, pre-registered via the Open Science Framework prior to data analyses (*link removed for blind peer review*), is a secondary analysis of data collected by Trickett et al. [50, 51], Agar [61] and Bissell et al. (unpublished data). These studies included cross-sectional actigraphy sleep assessments for children with SMS, AS, and TSC, as well as TD children, between September 2015 to September 2021. All children were aged between 4-15 years (inclusive), while the remaining eligibility criteria varied by group. Children with AS were included where (i) caregivers reported the child experienced sleep problems, and (ii) AS was attributed to *ubiquitin-protein ligase E3A* (UBE3A) deletion, to control for known phenotype differences between genotypes of AS [62]. In the SMS group, only children with current caregiver-identified sleep problems were included. Children with TSC were included regardless of caregivers’ perceptions of sleep problems. Confirmation of SMS and AS diagnoses were sought from caregivers^1^, and TSC diagnoses were confirmed via letters from healthcare professionals or saliva sample DNA tests. For the TD group, children were excluded where a statement of neurodevelopmental or health conditions, considered by a clinical psychologist to impact sleep, was provided (for example, ADHD). Children with genetic syndromes were recruited from syndrome support groups, social media, and databases of families who consented to contact regarding upcoming research studies at the University of Birmingham. Typically-developing children were recruited through social media, as well as family and friends of the research team. Ethical approval for the studies was obtained from the Science, Technology, Engineering and Mathematics Ethical Review Committee at the University of Birmingham.

For this secondary analysis, 20/31 children with SMS, 26/35 with AS, 16/22 with TSC and 61/70 TD children were included from the original studies. Five TD children were siblings of the children with rare genetic syndromes, and was therefore considered a predominantly independent sample. Children were excluded where an insufficient number of valid days of actigraphy data were available (< 5), as informed by previous research [55, 56, 64, 65]. Days were considered invalid where ≥ 3 hours of device removal was recorded [66, 67], where accompanying sleep diary data were unavailable, and/or where systematic errors in activity data were detected (e.g. constant activity across several days indicating no rest took place). For the majority of children, actigraphy data were collected on at least 4 weekdays and 1 weekend day, enhancing the representativeness of activity data which varies between weekdays and weekends [68, 69]. Children excluded from the secondary analysis did not substantially differ in age (*U* = 2061.00, *p =* 0.166), percentage of males (*χ*^2^(1) = 0.02, *p* = 0.893), or percentage of mobile children (*χ*^2^(1) = 0.68, *p* = 0.408) to those included. Similarly, within the syndrome groups, included and excluded children did not substantially differ across domains of adaptive functioning, including communication (*U* = 614.50, *p =* 0.444), daily living skills (*U* = 572.50, *p =* 0.783), socialisation (*U* = 539.50, *p =* 0.911), and motor skills (*U* = 478.00, *p =* 0.797). Additionally, minimal differences in age (*H*(3) = 3.18, *p=* 0.365) and percentage of males (*χ*^2^(3) = 4.90, *p* = 0.179) were found between the final groups included in the analysis. However, consistent with the physical phenotype reported in AS, the final AS group had a lower percentage of mobile children compared to the other groups (*χ*^2^(3) = 66.36, *p* < 0.001), and lower motor adaptive functioning relative to the final SMS (*U* = 32.00, *p* < 0.001) and TSC (*U* = 62.00, *p =* 0.015) groups. Greater communication (*U* = 37.00, *p* < 0.001), daily living (*U* = 52.50, *p* < 0.001), and socialisation skills (*U* = 110.50, *p =* 0.002) were also demonstrated in the final SMS group, compared to the final AS group. Table 1 describes the demographic characteristics and number of valid days of actigraphy data for each group included in the secondary analysis.

**Table 1:** Demographic characteristics and description of actigraphy data for the secondary analysis sample, separated by group

Group	Med age (Y) *(IQR)*	Male/ female (N)	Mobile/ immobile (N)	Med VABS Motor *(IQR)*	Med VABS Comm *(IQR)*	Med VABS Social *(IQR)*	Med VABS Daily *(IQR)*	Mean valid days of actigraphy data *(range)*	Children with actigraphy data on ≥4 weekdays and ≥1 weekend day (N)
AS	7.00*(6.38)*	10/16	10/15^a^	51.00*(5.00)*	43.50*(14.00)*	57.00*(13.25)*	43.00*(17.50)*	6.38*(5-11)*	24/26
SMS	8.50*(4.06)*	10/10	20/0	65.50^d^ *(12.25)*	67.00*(13.00)*	66.00*(19.00)*	66.00*(16.00)*	6.20*(5-9)*	20/20
TSC	8.45*(4.66)*	11/5	16/0	76.00^d^ *(38.50)*	58.00*(50.00)*	59.00*(40.00)*	63.50*(42.00)*	8.38*(5-9)*	15/16
TD	7.00^b^ *(4.75)*	36/24^c^	61/0	-^e^	-^e^	-^e^	-^e^	6.51*(5-9)*	61/61

*AS* Angelman syndrome, *Comm* communication domain, Daily daily living skills domain, *IQR* interquartile range, *Med* median, Motor motor skills domain, *SMS* Smith-Magenis syndrome, Social socialisation domain, *TSC* tuberous sclerosis complex, *TD* typically-developing, *VABS* Vineland Adaptive Behavior Scales, *Y* years

^a^Mobility data missing for one child with AS

^b^Age data missing for one TD child

^c^Sex data missing for one TD child

^d^Six children with TSC and one child with SMS did not receive motor skills scores as they exceeded the maximum age restriction for this domain

^e^VABS data were not collected for TD children

## Measures

### Actigraphy

All studies utilized the Actiwatch 2 (Philips Respironics), with all data downloaded and exported from Philips Actiware software. The devices were configured to the same settings across the studies, using a sampling rate of 32Hz and 30-second epochs. The Actiwatch 2 contains a solid-state uniaxial accelerometer that quantifies physical activity via activity counts [70]. The accelerometer detects the maximum acceleration of the device, from 0.5 to 2G, from 32 samples per second. This generates a maximum acceleration value per second which is added to an accumulating activity count; this recurs for as long as the predefined epoch length before the activity count is reset to 0 once the next epoch begins. The final activity count reflects the aggregated intensity and duration of movement during the epoch [71].

### Sleep diaries

Sleep diaries were completed by caregivers, either on paper [50, 51, 61] or via a mobile application (Bissell et al., unpublished data). All diaries had the same format, and prompted daily recordings of children’s sleep patterns and sleep-related behaviours, as well as timings of Actiwatch removal, daytime naps and evening sedentary activities (e.g. reading or watch television), to facilitate the removal of artifacts from actigraphy sleep estimates in the primary studies. The mobile application sleep diary also asked caregivers to rate children’s overactivity in the morning and evening of each day. Examples of the paper and mobile sleep diaries are available in Additional File 1: Attachment S1-S2.

### Background questionnaire

All studies obtained information regarding children’s age, sex, and genetic syndrome via a demographic questionnaire. This questionnaire also assessed children’s mobility, with children scored as “being able to walk unaided” or “not being able to walk unaided”.

### The activity questionnaire (TAQ [29])

All studies obtained subjective estimates of overactivity for children with SMS, AS and TSC via The Activity Questionnaire (TAQ). The TAQ contains 18 items that measure the frequency of behaviours associated with overactivity, impulsivity and impulsive speech within the last month. The frequency of each behaviour is indicated on a five-point Likert-type scale ranging from ‘Never/almost never’ (0) to ‘Always/almost all the time’ (4). The measure provides a total score and subscale scores for ‘overactivity’, ‘impulsivity’ and ‘impulsive speech’, with higher scores reflecting a greater frequency of the associated behaviours. The TAQ was developed for individuals of any age and intellectual ability, and has been employed frequently in research with rare genetic syndromes associated with intellectual disability [13, 19, 27]. The measure has good internal consistency (α = .77 - .95), and robust inter-rater (κ = .70 - .78) and test-retest reliability (*r* = .87 - .94) in rare genetic syndrome samples [29]. The measure also demonstrated good internal consistency across the current SMS (α = 0.88), AS (α = 0.89), and TSC (α = 0.87) groups.

### Vineland adaptive behavior scales (VABS [72, 73])

The VABS is a semi-structured interview designed to assess adaptive functioning in the domains of communication, daily living skills, socialisation, and motor skills. Adaptive functioning is a central component in the definition and measurement of intellectual disability [74], and was utilised as a proxy of intellectual disability severity in the current study. The VABS responses are summarized into age-equivalent and standardised scores, the latter of which are presented in the current study. Most studies ([50, 51, 61]) utilized the VABS Second Edition [73], and one study (Bissell et al., unpublished data) utilized the Third Edition [72].

### Procedure

Procedures varied slightly between the primary studies, complete details of each procedure are presented in the original works ([50, 51, 61], Bissell et al., unpublished data). Initially, a caregiver for each child completed the VABS interview in-person or during a telephone call. Each family then received an Actiwatch, sleep diary, and questionnaire pack. Children were asked to wear the Actiwatches continuously, either on the wrist or ankle, for between 7 and 10 days. Actiwatches were only to be removed during bathing, swimming or activities where the device may be damaged, such as during sport. Caregivers completed the sleep diaries for the duration that children wore the Actiwatches. The questionnaire pack was completed either before or during actigraphy data collection, via an online survey (Bissell et al., unpublished data) or paper handbooks [50, 51, 61]. All data were collected during school term time^1^ , although data for seven children with TSC and five TD children were collected during the 2020 COVID-19 national lockdowns. Within the TD group, modest evidence indicated greater M10 values for children assessed during the lockdowns compared to those assessed outside of lockdowns (*t*(59) = -1.35, *p* = 0.091). Within the TSC group, modest evidence of greater TAQ overactivity scores (*t*(14) = 1.64, *p* = 0.062) and strong evidence of greater M10 values (*t*(14) = 2.32, *p* = 0.018) was noted for children assessed during national lockdowns relative to those assessed outside lockdowns. Within the TD and TSC groups, weak-to-negligible differences in M10 onset were noted between children assessed during and outside national lockdowns (TD: *t*(58) = 1.09, *p* = 0.140; TSC: *t*(14) = -0.26, *p* = 0.401).

### Data selection and transformation

Several children had two actigraphy datasets as some primary studies were longitudinal [50, 51, 61]. In these cases, the analysed dataset was chosen following several criteria related to the number of days and ratio of weekdays to weekends (see complete criteria in Additional File 1: Figure S1). These criteria were designed to increase the reliability of children’s activity profiles, as children’s activity has been shown to vary considerably across consecutive days [33, 34], and between weekdays and weekends [68, 69].

**Fig.1:**
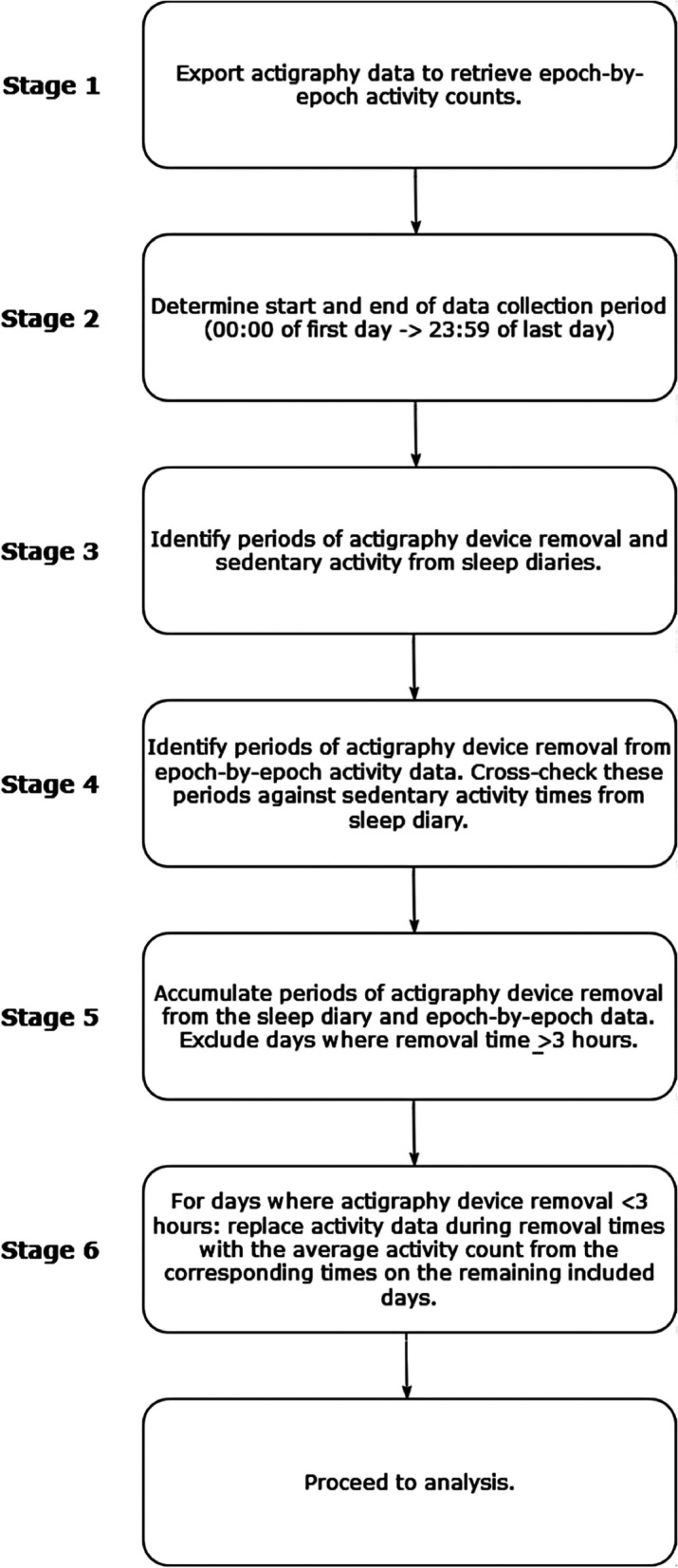
Summary of the cleaning protocol for actigraphy data

The actigraphy data were cleaned following a novel protocol developed by the primary author (ROS), briefly summarized in Figure 1 (see complete protocol and supporting citations in Additional File 1: Attachment S3). The protocol was designed following guidelines from previous research and primarily controlled for artifacts associated with Actiwatch removal, using data from sleep diaries. For example, ≥10 consecutive minutes of device-recorded inactivity during wakeful periods was identified as indicative of device removal, where this did not overlap with sedentary periods in sleep diaries. Additionally, ≥120 consecutive minutes of device-recorded inactivity during sleep was indicative of device removal. Activity data during periods of device removal were substituted with an average activity count, collated from the remaining days at the corresponding time. Where ≥3 hours of device removal was recorded within a single day, this day was excluded from the analysis. The protocol ensured the analysed data were representative of children’s typical activity levels.

All actigraphy data were cleaned by a single researcher (ROS), and ≥25% of data were cleaned by a second researcher (AK) at each stage of the cleaning protocol. Inter-rater reliability analyses revealed excellent agreement at all stages of data cleaning: stage 3 (ICC = 0.99, 95% CI [0.99; 0.99]), stage 4 (ICC = 0.99, 95% CI [0.99; 0.99]), stage 5 (ICC = 0.96, 95% CI [0.94; 0.97]) and stage 6 (ICC = 0.98, 95% CI [0.98; 0.98]). Discrepancies were discussed between the authors until consensus was reached.

### Analyses

Prior to statistical analyses, the non-parametric circadian rhythm variables M10 and M10 onset time were calculated for each child using the *pyActigraphy* package for Python V3.8.0 [75]. The M10 reflects the average activity count, per epoch, during the most active 10 hours of the day. This 10-hour period is selected from a child’s average 24-hour activity profile, collated from several days of actigraphy data. The M10 reflects activity intensity during the most active hours of the day, and is consistently elevated in clinical populations associated with overactivity compared to TD peers [45–49]. Therefore, this variable was considered to maximise the sensitivity of actigraphy-defined movement to overactivity. The time at which the most active 10-hour period started within the averaged 24-hour profile was also recorded as M10 onset.

Unless otherwise specified, all statistical analyses were performed in IBM SPSS version 29. The assumptions of parametric tests were verified prior to analysis, and non-parametric tests were utilized when these assumptions were violated, given the small group sizes [76]. One outlier was identified in the TD group for M10 onset time (13:38:00) and was thus removed from the M10 onset analyses. Although included in the study pre-registration, the within-group receiver operating characteristic curve analyses were not conducted as the group samples were too small for sufficient power [77].

To first determine whether children’s mobility levels impacted overactivity estimates, exploratory independent samples t-tests were conducted within groups to explore differences in TAQ overactivity, M10, and M10 onset between children who could and could not walk unaided. The mobility analyses were limited to AS since all children in the remaining groups could walk unaided.

To examine the convergence of actigraphy-defined activity and questionnaire overactivity estimates, Pearson’s r correlations were calculated between M10/M10 onset and TAQ overactivity subscale scores for each group. Where significant or non-negligible correlations were observed (r > .1), linearity was visually examined using scatterplots. Functional linear modelling (FLM) and non-parametric permutation F-tests were conducted to explore differences in average 24-hour activity profiles between children with high and low TAQ scores, identified via median split. The respective medians for the SMS, AS, and TSC groups were 24, 16, and 22. These analyses were conducted using the *Actigraphy* R package [78]. Within FLM, the average 24-hour activity profiles are plotted for each group by converting raw activity data to a functional form, using a Fourier expansion model fitted at a 24-hour periodicity. The Fourier expansion included 9 basis functions as this captures major activity trends with limited noise [65]. Minute-by-minute differences in 24-hour activity profiles between syndrome and TD groups were explored using non-parametric permutation F-tests, entailing 5000 permutations as recommended for small group samples [79]. For these tests, significance is calculated by counting the proportion of permutation *F* values greater than the *F-*statistics of the compared groups. The maximum critical value is a single number reflecting the proportion of maximised *F-*values from each permutation, whereas the point-wise critical value is a curve reflecting the proportion of all permutation *F* values at each point in the time series. The maximum critical value is considered more robust [65], and was thus used for the current study. Additionally, to determine whether between-group differences reflect differences in wakeful activity, the timings of between-group differences were compared against mean sleep onset and offset times for each group, previously calculated with actigraphy ([50, 51, 61], Bissell et al., unpublished data). Sleep onset time indicates the beginning of the first period sleep in a given day, whereas sleep offset time marks the end of the final period of sleep in a given day.

To examine differences in actigraphy-defined activity between the syndrome and TD groups, M10 and M10 onset data were compared between the groups using Kruskal-Wallis tests and follow-up Dunn-Bonferroni pairwise comparisons. Additional FLMs and non-parametric permutation F-tests were conducted, following the same specifications as the initial FLMs, to explore between-group differences in 24-hour activity profiles. The timings of group differences were also compared with previously-calculated actigraphy mean sleep onset and offset times for each group ([50, 51, 61], Bissell et al., unpublished data).

To control for inflated type I error from multiple comparisons, *p-*values were corrected via the Bonferroni adjustment. Two separate adjustments were applied to the tests addressing the first (0.05/9 = 0.006) and second (0.05/5 = 0.01) study aims, as the underlying aims of these tests were considered distinct enough to form two families of tests [80]. Results that meet these thresholds are termed ‘significant’ throughout the paper. Exceptions to these corrections included results from Dunn-Bonferroni post-hoc tests as these already control for type I errors [81]. To mitigate against type II error arising from the small, clinical sample, *p*-values that did not meet significance thresholds were interpreted, in conjunction with effect sizes, as graded evidence. More specifically, smaller *p*-values observed alongside larger effects more greatly refute the null hypothesis, compared to larger *p*-values observed alongside smaller effects [82–84]. This interpretation of *p*-values has received increased support in recent years, as a means of tackling replicability issues and oversimplified acceptance/rejection of null hypotheses based on significance thresholds [82]. Following this interpretation of *p*-values, omnibus tests that had sufficiently small *p-*values (<0.15) were explored further with post-hoc tests.

To determine whether the COVID-19 national lockdowns affected the study findings, all analyses were repeated after excluding those TD children recruited during the lockdowns. This was not repeated for the TSC group as a substantial proportion of this small group were recruited during the lockdowns (7/16). The results of these analyses are referenced throughout the paper, with respective tables and figures presented in Additional File 1: Table S1, Figures S2-S4.

## Results

Exploratory analyses were first conducted to determine whether mobility levels influenced actigraphy-defined activity and TAQ overactivity scores. No *p-*values met significance thresholds, however a small *p*-value and modest *t*-statistic suggested that M10 values may be greater for children with AS classified as “being able to walk unaided” than those “not being able to walk unaided” (Table 2). A large *p*-value and near-zero *t*-statistic highlighted little-to-no difference in M10 onset between the mobility groups. A moderately small *p*-value and modest *t*-statistic suggested TAQ overactivity scores may have been larger for the children “able to walk unaided” compared to those who could not.

**Table 2:** Mean scores for actigraphy and questionnaire variables, for children with AS, and between-mobility group comparisons

	Group		Between-group differences
Variable	AS walk unaided (*n*= 10)	AS cannot walk unaided (*n*= 15)	*p* value
Mean M10 *(SD)*	322.87*(106.32)*	241.68*(105.91)*	0.037
Mean M10 onset *(SD)*	8:59:06*(80.50)*	9:01:40*(66.93)*	0.466
Mean TAQ overactivity *(SD)*	24.11^a^ *(5.09)*	19.73*(9.21)*	0.103

*Abbreviations. AS* Angelman syndrome, *TAQ* The Activity Questionnaire

Mobility data was missing for one child in AS group, excluded from this table and between-group analyses

^a^TAQ data missing for one child in AS mobile group

**Fig. 2:**
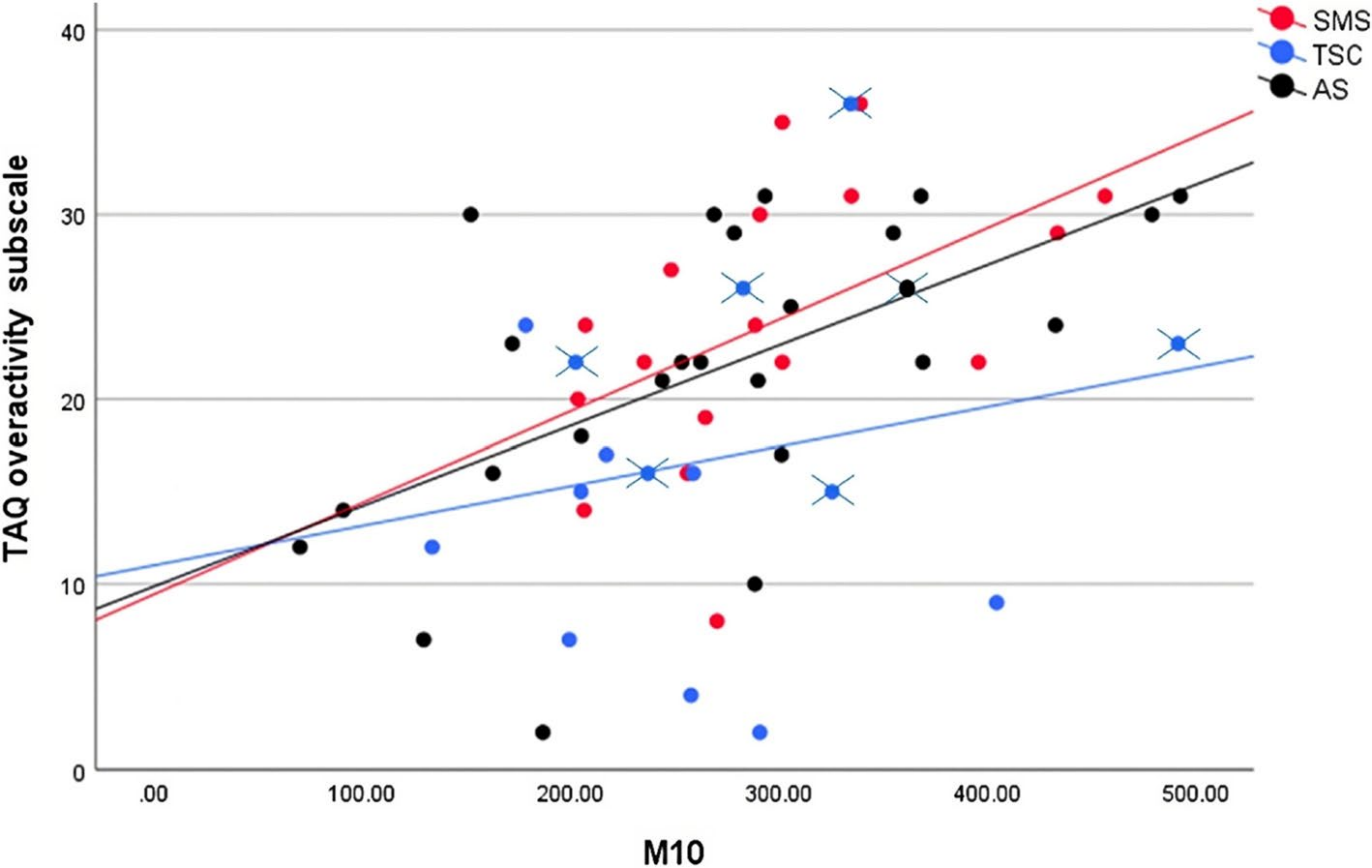
Scatterplots for M10 and TAQ overactivity data with lines of best fit, for each syndrome group Data points with crosses represent children recruited during COVID-19 national lockdowns

To address the first aim of the study, the convergence of actigraphy-defined activity and TAQ overactivity subscale scores was assessed. One child in the AS group and three children in the SMS group were excluded from these analyses due to missing TAQ data. In contrast, a significant, large correlation was demonstrated between M10 and TAQ overactivity scores in the AS group (*r* = 0.598, *p* = 0.002). A small *p-*value and large correlation also supported the relatedness of the M10 and TAQ data in the SMS group (*r* = 0.499, *p* = 0.041), whereas a large *p-*value and small correlation refuted the relatedness of these variables in the TSC group (*r* = 0.218, *p* = 0.416). In addition, large *p*-values and small-to-negligible correlations challenged associations between M10 onset time and TAQ overactivity scores across all syndrome groups (see Additional File 1: Table S2). The linearity of M10 and TAQ overactivity scores was further visually examined via scatterplots, presented in Fig. 2. The AS plot revealed strong linearity, with greater dispersion from the line of fit for M10 values ≤ 300. The SMS plot demonstrated less variation in M10 values, although strong linearity and relatively consistent dispersion from the line of fit was observed. The TSC plot revealed poor linearity, with wide dispersion from the line of fit.

**Fig. 3:**
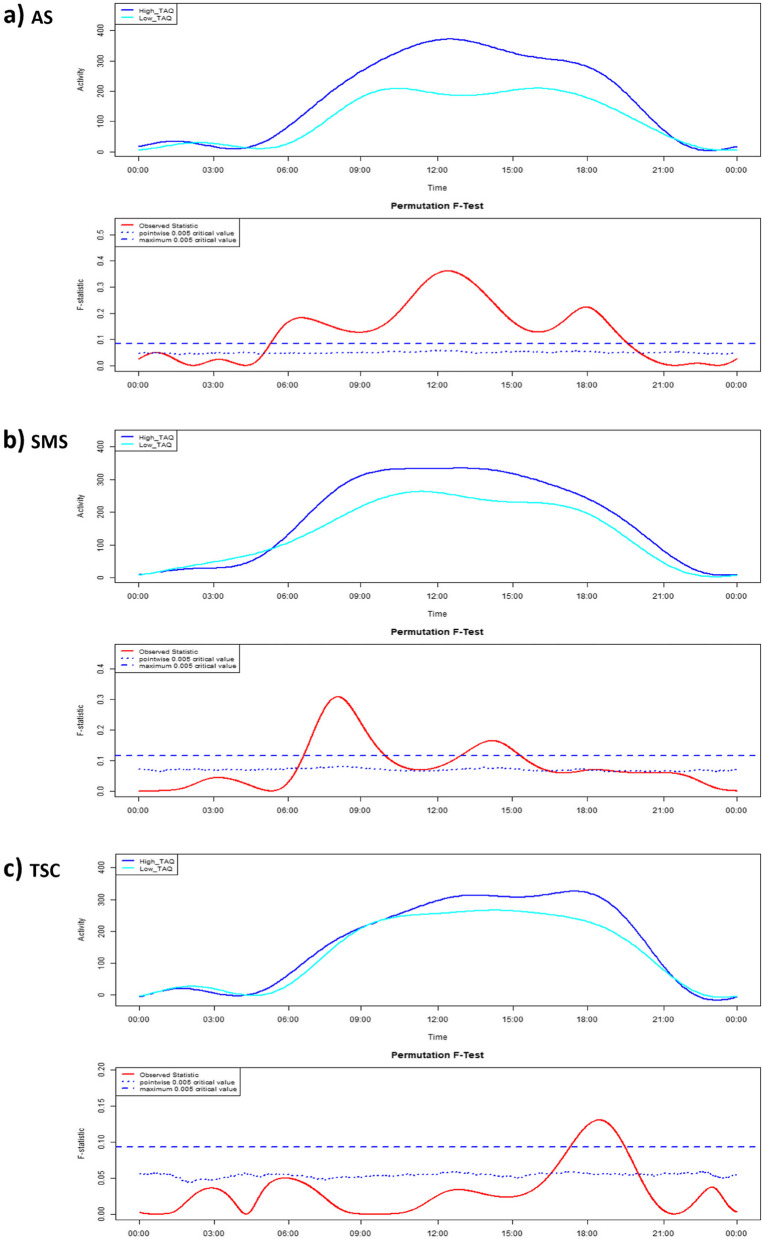
FLMs and permutation F-tests comparing 24-h activity profiles between high and low TAQ overactivity groups Upper panels for each syndrome present the mean 24-hour activity profile of the high and low TAQ groups, converted to functional form via Fourier expansion model. Lower panels present the permutation F-test output. Results were considered significant where the observed statistic (red line) is greater than the maximum critical value (horizontal dashed blue line). **a** Angelman syndrome, **b** Smith-Magenis syndrome, **c** tuberous sclerosis complex

To further examine the associations between actigraphy-defined activity and TAQ overactivity scores, 24-hour activity profiles were generated via FLM for children with high and low TAQ overactivity within each syndrome. Differences in activity profiles were explored via non-parametric permutation F-tests. The results of these tests for AS, SMS and TSC groups are presented in Fig. 3. For the AS group, significantly higher activity was observed for the high TAQ group compared to the low TAQ group from approximately 05:20 – 19:30; with between-group differences in activity peaking during early morning, midday and evening hours. For the SMS group, significantly higher activity was observed in the high TAQ group compared to the low TAQ group from approximately 06:45 – 10:00, and 13:00 – 15:30. For the TSC group, significantly higher activity was observed in the high TAQ group compared to the low TAQ group from approximately 17:30 – 19:30.

Activity differed between the high and low TAQ groups during daytime hours between the mean sleep offset and onset times for each group, aside for AS. For the AS group, differences in activity preceded the mean sleep offset times for both the high (06:10:04) and low (07:01:05) groups.

To address the second aim of the study, between-group differences in M10 and M10 onset were explored between all groups (Table 3). Kruskal-Wallis tests revealed a moderately small *p*-value for M10 comparisons (*H*(3) = 5.85, *p =* 0.119) although post-hoc Dunn-Bonferroni pairwise comparisons produced large *p*-values (*p* = 0.335 – 1.00), indicating no credible between group differences. A small *p*-value and relatively larger effect was observed for M10 onset comparisons (*H*(3) = 7.78, *p* = 0.049), and post-hoc Dunn-Bonferroni pairwise comparisons indicated earlier M10 onset in the SMS group compared to the TD group (*p* = 0.037). The outcomes of these tests did not meaningfully change following the exclusion of those TD children recruited during COVID-19 lockdowns (see Additional File 1: Table S1).

**Table 3:** Median M10 and M10 onset scores for each group, alongside between-group comparisons and post-hoc tests

	Group				Between-group comparison	
Variable	AS	SMS	TSC	TD	*p* value	Post-hoc test
Median M10 *(IQR)*	283.47*(173.46)*	279.49*(99.36)*	258.35*(129.07)*	330.08*(147.83)*	0.119	-
Median M10 onset *(IQR)*^a^	08:52:00*(98.50)*	08:20:00*(102.75)*	09:01:30*(117.25)*	09:19:00^b^ *(135.00)*	0.049	SMS earlier than TD

*Abbreviations. AS* Angelman syndrome, *IQR* interquartile range, *SMS* Smith-Magenis syndrome, *TD* typically-developing, *TSC* tuberous sclerosis complex

^a^Interquartile range presented in minutes

^b^M10 onset descriptive statistics following the exclusion of one outlier

**Fig. 4:**
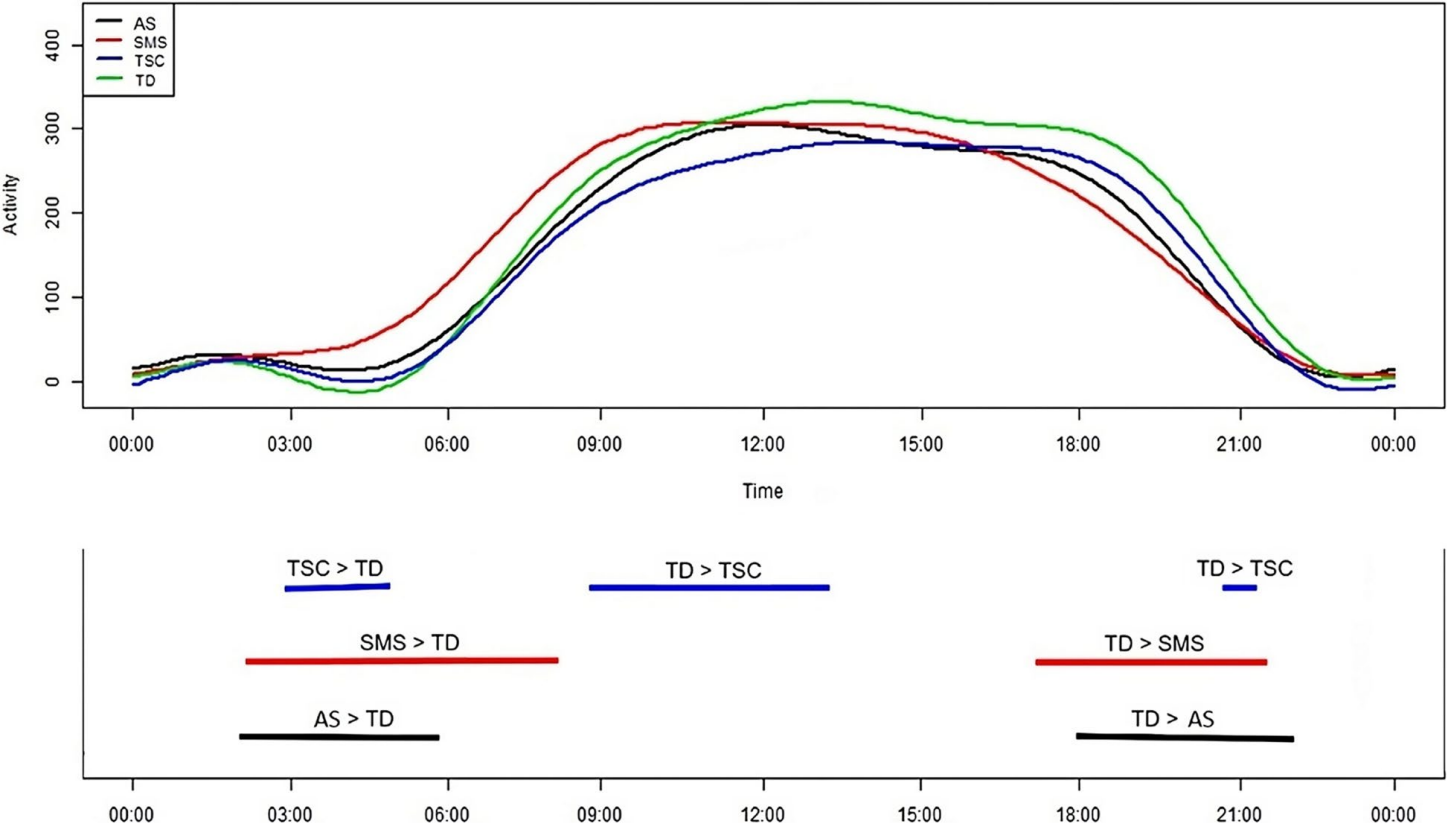
FLMs and permutation F-tests comparing 24-hour activity profiles between typically-developing and syndrome groups Upper panel presents the mean 24-hour activity profile of each group, converted to functional form via Fourier expansion model. Lower panel presents the non-parametric permutation F-test results. The lines correspond to the times at which activity levels significantly differed between the syndrome and TD groups (*p* = .01), and describe the direction of the effect

Additionally, differences between 24-hour activity profiles of TD and syndrome groups were explored using FLM and non-parametric permutation F-tests. The combined results of these tests are presented in Fig. 4, while FLMs and permutation F-tests for specific TD-syndrome comparisons are presented in Additional File 1: Figures S5-S7. Compared to the TD group, activity was significantly higher in: 1) the TSC group from approximately 02:50 – 05:00, 2) the SMS group from approximately 02:15 – 08:15, and 3) the AS group from approximately 02:00 – 05:50. Comparisons with the TD group also revealed significantly lower activity in: 1) the TSC group from approximately 08:40 – 13:15 and 20:45 – 21:15, 2) the SMS group from approximately 17:00 – 21:30, and 3) the AS group from approximately 18:00 – 22:00.

Following the exclusion of TD children recruited during COVID-19 lockdowns, instances of heightened activity in the TD group increased compared to the AS and TSC groups (see Additional File 1: Figures S2 and S4). Compared to the TD group, activity was significantly lower in: 1) the AS group from approximately 08:00 – 08:45, and 13:30 – 22:00, and 2) the TSC group from approximately 06:45 – 15:00, and 19:30 – 21:30. No change was observed for TD-SMS comparisons.

Instances of heightened activity amongst the syndrome groups, relative to the TD group, frequently preceded the mean sleep offset times of the TSC (06:54:22), SMS (05:02:18), AS (06:31:54), and TD (07:03:40) groups. Additionally, the times of elevated activity amongst the TD group, compared to the syndrome groups, often surpassed the mean sleep onset times for the TSC (20:28:04), SMS (20:04:47), AS (20:11:20), and TD (21:15:12) groups.

## Discussion

This study is the first to examine the sensitivity of actigraphy to overactivity in children with rare genetic syndromes, via comparisons with TD peers and overactivity estimates from established questionnaire techniques. This study is also the first to use actigraphy to delineate the temporal characteristics of heightened physical activity in children with rare genetic syndromes across 24 hours. The findings are strengthened by: (i) multi-day actigraphy assessments in children’s naturalistic environments, rigorous data cleaning procedures, and strict inclusion criteria that enhance the representativeness of children’s activity profiles; (ii) use of the TAQ, the psychometric properties of which have been established in rare genetic syndrome samples [29]; and (iii) inclusion of a TD comparator group containing few siblings, reducing biases in the syndrome-TD comparisons.

The first aim of the study was addressed by exploring correlations between TAQ overactivity estimates and M10 within each syndrome group. Syndrome-specific patterns of linear correlation were observed between TAQ overactivity and M10 estimates, with large positive correlations observed in AS and SMS, and a weaker correlation in TSC. These results partially contradict previous findings, demonstrating weak and/or non-significant correlations between questionnaire overactivity estimates and actigraphy data amongst overactive children [39, 40, 85]. Nevertheless, for children with AS and SMS, the findings provide preliminary support for the convergent validity of actigraphy-defined activity, isolated during the most active 10 hours of the day, and TAQ overactivity estimates. This indicates that prolonged instances of heightened activity, spanning 10 hours, may capture the same overactivity construct as perceived by caregivers of children with AS and SMS. Conversely, poorer convergent validity of M10 and TAQ data for children with TSC indicates that overactivity in these groups, as perceived by caregivers, is not as closely associated with heightened activity spanning ~10 hours. The results should be interpreted cautiously and affirmed with a larger sample, given the small group sizes and limited variance of M10 and TAQ values.

Average 24-hour activity profiles were also compared between children with high and low TAQ overactivity scores, within each syndrome group. In contrast to the M10-TAQ comparisons, the results support the convergent validity of actigraphy and TAQ data for all syndromes, as children with high TAQ scores were often more active throughout the 24-hour cycle compared to those with low TAQ scores, and never less active. Indeed, the convergence of TAQ and actigraphy data suggests that periods of heightened activity in the high TAQ groups likely reflect overactivity, as perceived by caregivers. Syndrome-specific differences were observed in the timing, frequency and duration of heightened activity in the high TAQ groups, suggesting that overactivity may manifest distinctly between genetic syndromes. For example, heightened activity of the high TAQ groups was observed: (i) throughout the early morning until late evening for children with AS, (ii) during the early morning and early afternoon for children with SMS, and (iii) during the early evening for children with TSC. These syndrome-specific temporal descriptions of overactivity are completely novel, advancing existing description provided by questionnaire techniques. The syndrome-specific presentations of overactivity may underpin between-syndrome differences in M10-TAQ overactivity correlations. Indeed, the strongest correlation was observed for AS where overactivity was sustained throughout the day; a slightly less strong correlation was demonstrated for SMS where overactivity presented in bursts; and a relatively weak correlation was found for TSC where overactivity presented briefly in the evening. However, the novelty of the findings necessitates replication, and future studies should recruit larger samples given the limited size of the current high and low TAQ groups.

In most instances, differences in activity between the high and low TAQ groups occurred between sleep offset and onset times, further suggesting that heightened activity amongst the high TAQ groups is indicative of wakeful overactivity. An exception to these findings was observed for the AS group, where greater activity was demonstrated in the high TAQ group relative to the low TAQ group from approximately 05:20:00. This finding may be explained as 5/15 children in the high TAQ group had mean sleep offset times within +/- 15 minutes of 05:20:00, whereas the earliest individual mean sleep offset time in the low TAQ group was 06:02:20. Therefore, heightened activity amongst the high TAQ group may be driven by the greater likelihood of wakefulness, which increases activity relative to sleep. Given the specificity of the TAQ to overactivity, heightened activity ~05:20:00 in the high TAQ group may reflect wakeful overactive behaviour. However, this finding may also be driven by wakeful non-overactive behaviours. Early morning wakeful activity, even when non-overactive, may disturb caregivers’ sleep and be considered challenging, and therefore may incur more severe subjective ratings of overactivity. Future studies should examine the qualitative characteristics of wakeful early morning behaviours amongst children with rare genetic syndromes, in order to identify the presence of early morning overactivity.

Instances of heightened activity in the high TAQ groups, relative to the low TAQ groups, may also be underpinned by syndrome-specific characteristics, providing insights into the factors that may influence caregivers’ appraisal of children’s overactivity. For example, for the AS group, persistent heightened activity for children with high TAQ scores may be driven by the higher proportion of ambulatory children in the high TAQ group (7/14) compared to the low TAQ group (2/10), as mobility impacts actigraphy-defined activity levels [86]. For children with SMS, elevated early morning activity in the high TAQ group may be underpinned by a phase-advanced circadian rhythm and accompanying early morning awakenings, characteristic of the syndrome [19, 87]. Early morning awakenings may incur wakeful early morning activity, which may influence caregivers’ perceptions of overactivity given the challenging nature of children’s activity at this time of day. Differences in early afternoon activity appear driven by reductions in activity within the low TAQ group, the timing of which coincides with preferred napping time in SMS (12:00 – 15:00, [51, 88]). Therefore, caregivers’ perceptions of overactivity in SMS may also be influenced by children’s napping propensity. For the TSC group, heightened evening activity in the high TAQ group aligns with caregivers’ overactivity ratings on the mobile application sleep diary, wherein overactivity was recorded on 42 evenings across the high TAQ group, compared to 7 evenings in the low TAQ group. These corroborating data suggest that children’s overactive behaviours in the evening may influence caregivers’ global estimates of children’s overactivity. Overall, syndrome-specific characteristics that affect children’s activity levels at different times throughout the day may impact caregivers’ overall appraisal of children’s overactivity. Future studies should directly examine the effect of syndrome-related characteristics on actigraphy-defined activity and caregivers’ overactivity ratings.

The second aim of the study was addressed via M10 comparisons between syndrome and TD groups, the results of which opposed between-group differences. This demonstrates that the magnitude of activity, averaged across the most active 10 hours of the day, did not considerably differ between genetic syndromes associated with overactivity and TD peers. This is in contrast to evidence of greater M10 values in other overactive populations, namely ADHD, compared to TD controls [46–49]. The inconsistent findings may be underpinned by the variability of overactivity within rare genetic syndrome groups, precluding the detection of overactivity in group-level data, unlike in ADHD for which overactivity constitutes diagnostic criteria [1, 2]. The variability of overactivity is reflected by the broad dispersion of M10 and TAQ overactivity scores within the syndrome groups (see Fig. 2), as well as previous prevalence estimates indicating that between 25-50% of children in the investigated syndromes may not be overactive [4, 6, 7]. Alternatively, the dispersion of M10 values may be driven by syndrome-related characteristics that impact activity, and vary between individuals. Such characteristics may include nap frequency and length [3], daytime sleepiness [89], interest and pleasure engaging in activities [27, 90], and mobility levels [91–93]. Indeed, evidence of greater M10 values was observed for children with AS classified as ambulatory, compared to those classified as non-ambulatory. To further understand the sensitivity of M10 to overactivity in children with rare genetic syndromes, future research should examine the impact of syndrome-related characteristics on M10 data, and explore differences in M10 between children classified by caregivers as overactive and non-overactive.

The between-group comparisons also provided evidence for earlier M10 onset for children with SMS compared to TD children. This indicates that, despite no substantial between-group differences in the magnitude of activity during the most active 10 hours of the day (i.e. M10), this 10 hour period may start earlier in the day for children with SMS relative to TD peers. This finding aligns with reports of early morning awakenings in SMS [51, 94], likely leading to earlier increases in activity. However, given the preliminary nature of the results, future studies should re-examine these group differences with larger samples.

Average 24-hour activity profiles were also compared between the syndrome and TD groups, revealing syndrome-specific patterns of physical activity. There was also evidence of greater activity during early morning hours for the syndrome groups compared to TD peers. For all syndrome groups, high early morning activity preceded the mean sleep offset times, indicating heightened activity occurred during sleep periods. These findings may be explained by the high frequency and duration of night awakenings in each syndrome [19, 50, 51, 95, 96]. Night awakenings may increase opportunities for wakeful overactivity, as has been reported in SMS [97], although they can enable other non-overactive behaviours that increase activity relative to sleep (e.g. playing with toys or rocking [98]). Heightened activity in the SMS and AS groups may also occur during sleep given the prevalence of periodic limb, hyperkinetic, and pain-related movements in AS [98–101], as well as sleep enuresis in AS and SMS which can reduce the proportion of motionless sleep [89, 102]. Furthermore, whilst heightened activity in the SMS group persisted for approximately 3 hours following the mean sleep offset time (~05:02:00 – 08:15:00), the TD mean sleep offset time occurred later in the morning (07:03:40). Therefore, from ~05:00:00 – 07:00:00, higher activity in the SMS group compared to the TD group may not reflect overactivity, but simply greater activity driven by wakefulness relative to sleep. As such, across the syndrome groups, it is unclear whether heightened early morning activity reflects wakeful overactivity. Future research should directly examine the times of night awakenings and presence of wakeful overactivity at night, and thus determine whether instances of heightened activity during early morning hours reflect wakeful overactivity or not.

Comparisons of average 24-hour activity profiles also revealed that, after early morning hours, activity levels either did not differ between the syndrome and TD groups, or were lower in the syndrome groups. This corresponds with previous visual comparisons of 24-hour activity levels between children with SMS and TD siblings [58], but is otherwise novel. As mentioned for the M10 results, the lack of group differences may be explained by the variability of overactivity within syndrome groups, hindering the detection of overactivity within the pooled activity data. Instances of lower activity in the syndrome groups, relative to the TD group, may also be explained by syndrome-related characteristics. For example, lower activity in the TSC and SMS groups may be driven by excessive daytime sleepiness, as this occurs in approximately 46% and 60% of individuals, respectively [89], and is associated with reduced physical activity [103, 104]. Lower activity throughout the early evening in the syndrome groups may be underpinned by limited opportunities to engage with after-school programmes and sports, as previously observed for children with intellectual disabilities [105, 106]. Additionally, lower activity in the syndrome groups from later evening to nocturnal hours may be driven by earlier bedtimes relative to TD peers, as evidenced by the current sleep onset times and previous findings [50, 51, 107]. Physical activity is limited whilst in bed, and is also typically reduced during bedtime routines [108]. Future research should directly examine the impact of syndrome-related characteristics on actigraphy-defined activity throughout the day.

Despite the results of the syndrome-TD comparisons, the M10-TAQ correlations and high-low TAQ group comparisons broadly support the convergent validity of actigraphy-defined activity and TAQ overactivity estimates. This supports the sensitivity of actigraphy to subjectively-defined overactivity reported by caregivers of children with rare genetic syndromes, and suggests that researchers should further examine the properties of actigraphy as a measure of overactivity in this population. Actigraphy offers several advantages for measuring overactivity, including: (i) broad tolerance amongst children with rare genetic syndromes [50–53], (ii) resistance to subjective biases, (iii) sensitivity to children’s naturalistic activity profiles, and (iv) an ability to estimate children’s 24-hour activity levels, from which temporal properties of overactivity can be inferred. These attributes may promote the rigour, precision and representativeness of overactivity estimates, relative to traditional questionnaire techniques. Additionally, examining the temporal properties of overactivity can advance descriptions of this behaviour within syndromes and, in turn, provide opportunities to deepen existing understanding of relationships between overactivity and other clinically-significant behaviours. For example, whilst overactivity has been associated with sleep disturbances in rare genetic syndromes [18, 19], overactivity in the evening may incur sleep disturbances, whereas overactivity in the morning may result from sleep disturbances. However, despite the advantages of actigraphy, questionnaire techniques should not be discarded. Indeed, the convergence of questionnaire overactivity estimates with actigraphy data, that directly reflects activity levels, in the current study also supports the validity of questionnaire techniques. Unlike actigraphy, questionnaires assess the qualitative characteristics of heightened physical activity, such as difficulty holding still or boisterous engagement with activities [29], and measure aspects of overactivity other than heightened physical activity, such as fidgeting with objects or discomfort staying still [1, 2]. Therefore, as similarly noted in sleep research [109], questionnaire and actigraphy techniques provide complementary information necessary to comprehensively examine overactivity in children with rare genetic syndromes.

In drawing conclusions from this study, several limitations of the actigraphy data should be considered. Firstly, the current findings are based on children from the United Kingdom, however children’s activity profiles may differ between cultures given different school regimes and social practices [110, 111]. Future research should examine the cross-cultural generalisability of the current findings. Additionally, the data was collected by several Actiwatch 2 devices, yet the reliability of accelerometer readings and activity counts between devices was not assessed or corrected for prior to data collection, possibly confounding within-group activity profiles and between-group differences. Despite this, no previous actigraphy studies that assessed overactivity addressed this issue (e.g. [64, 112, 113]), and evidence indicates excellent inter-device reliability for Actiwatch 2 activity counts [114]. Instances of external motion, such as being in a car, can bias actigraphy activity data but were also not assessed or controlled for [115]. Future studies should control for artifacts associated with external motion when examining overactivity with actigraphy. Furthermore, activity data for the TSC group seemed impacted by the COVID-19 national lockdowns but could not be corrected for, reducing the representativeness of the TSC group-level data. Despite this, the sensitivity of actigraphy to overactivity amongst these children was still robustly assessed via comparisons with TAQ overactivity scores, also collected during national lockdowns. Activity amongst the AS group may have also been biased by the sole inclusion of children with UBE3A deletion, whose mobility is developmentally delayed relative to those with other AS genotypes [62]. In addition, activity of the AS and SMS samples may have been skewed as the inclusion criteria required children to experience sleep difficulties, which are positively associated with overactivity [15, 18, 19]. Despite this bias, the findings retain broad representativeness as sleep difficulties are prevalent amongst AS and SMS populations, occurring in approximately 70% and 95% of individuals, respectively [89]. Although children diagnosed with neurodevelopmental/health conditions, such as ADHD, were excluded from the TD group, no additional screening of overactivity or overactivity-associated conditions was completed. To minimize threats to internal validity, future studies that examine the sensitivity of actigraphy to overactivity should employ more rigorous screening procedures for non-overactive comparison groups. Finally, ≥25% of children within each syndrome group were excluded from the current analysis because Actiwatch devices were worn for an insufficient length of time, limiting the generalizability of the activity data to a subset of children within each syndrome group who can tolerate actigraphy for several days. Despite these limitations, this study is the first to highlight the potential utility of actigraphy for evaluating overactivity in children with rare genetic syndromes. Future studies should address the current limitations, and thus obtain more rigorous insights into the sensitivity of actigraphy to overactivity in rare genetic syndromes.

## Conclusion

This study is the first to directly examine the sensitivity of actigraphy to overactivity in children with rare genetic syndromes, and utilise actigraphy to describe activity profiles and overactivity in these populations. Although activity levels were often similar or lower in the syndrome groups relative to TD peers, good convergent validity was indicated across syndrome groups via comparisons between actigraphy-defined activity and questionnaire overactivity estimates. As such, future research should continue to examine the properties of actigraphy as a measure of overactivity, by replicating this study with larger samples, examining the effect of syndrome-related characteristics on actigraphy-measured activity, and investigating the sources of heightened activity occurring during sleep periods.

### Supplementary Information

**Supplementary Material 1.**

## Data Availability

The datasets analysed in the current study are available from the corresponding author, upon reasonable request.

## Abbreviations

ADHD: attention-deficit hyperactivity disorder
AS: Angelman syndrome
FLM: Functional linear model
SMS: Smith-Magenis syndrome
TAQ: The Activity Questionnaire
TD: typically-developing
TSC: tuberous sclerosis complex

## Footnotes

1 Several methodological details are not reported in the published primary studies (Trickett et al., [50]; Trickett et al., [51]), but are reported in the corresponding doctoral thesis [63]. Therefore, the doctoral thesis may be referenced when describing the study methodologies.
