# Supplementary material for: Exploring an objective measure of overactivity in children with rare genetic syndromes
Source: J Neurodev Disord. 2024 Apr 18;16:18. doi: 10.1186/s11689-024-09535-y (PMC11025271; doi:10.1186/s11689-024-09535-y)
Supplement: Supplementary file 1 — Supplementary Material 1. [file 11689_2024_9535_MOESM1_ESM.docx]

| **Figures/Attachments** | **Content** |
| --- | --- |
| Attachment S1 | Paper sleep diary example |
| Attachment S2 | Mobile sleep diary example |
| Figure S1 | Flow diagram for choosing the dataset to analyse |
| Attachment S3 | Actigraphy data cleaning protocol |
| Table S1 | Median scores for M10 and M10 onset for each group, alongside between-group comparisons and post-hoc tests, following the exclusion of TD children recruited during COVID-19 national lockdowns. |
| Figure S2 | Functional linear model and permutation F-test for 24-hour activity profiles of children with Angelman syndrome and typically-developing children, following exclusion of the TD children recruited during COVID-19 national lockdowns. |
| Figure S3 | Functional linear model and permutation F-test for 24-hour activity profiles of children with Smith-Magenis syndrome and typically-developing children, following exclusion of the TD children recruited during COVID-19 national lockdowns. |
| Figure S4 | Functional linear model and permutation F-test for 24-hour activity profiles of children with tuberous sclerosis complex and typically-developing children, following exclusion of the TD children recruited during COVID-19 national lockdowns. |
| Table S2 | Correlations between M10 onset and TAQ overactivity subscale scores, across syndrome groups. |
| Figure S5 | Functional linear models and permutation tests for comparisons of 24-hour activity profiles between typically-developing and Angelman syndrome groups |
| Figure S6 | Functional linear models and permutation tests for comparisons of 24-hour activity profiles between typically-developing and Smith-Magenis syndrome groups |
| Figure S7 | Functional linear models and permutation tests for comparisons of 24-hour activity profiles between typically-developing and tuberous sclerosis complex groups |

**Table of contents**

Additional File 1: **Attachment** **S1:** Paper sleep diary example

| **To be completed throughout the day** | | | | | | | | | **Completed by (initials)** |
| --- | --- | --- | --- | --- | --- | --- | --- | --- | --- |
| Time Actiwatch Removed |  | | | Time Actiwatch Replaced | | | |  |  |
| Time Actiwatch Removed |  | | | Time Actiwatch Replaced | | | |  |  |
| Time Actiwatch Removed |  | | | Time Actiwatch Replaced | | | |  |  |
| **To be completed in the evening** | | | | | | | | | |
| **Nap 1**  Start time:  End time: | **Nap 2**  Start time:  End time: | | | | | **Nap 3**  Start time:  End time: | | |  |
| **Please list any sedentary activities after 6pm e.g. reading alone or with an adult, watching TV** | | | | | | | | | |
| **Type of activity (select one)**  Watching TV  Reading alone or with an adult  Other- please state | | **Start time of activity** | | | | | **End time of activity** | |  |
| **Type of activity (select one)**  Watching TV  Reading alone or with an adult  Other- please state | | **Start time of activity** | | | | | **End time of activity** | |  |
| **Type of activity (select one)**  Watching TV  Reading alone or with an adult  Other- please state | | **Start time of activity** | | | | | **End time of activity** | |  |
| **Time got into bed:** | | **Was proximity watch put on?**  **Yes No** | | | | | | |  |
| **Time lights turned off:**  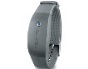 | | | | | | | | |  |
| **To your knowledge, was the event marker pressed at the correct time? (Please circle) Yes No** | | | | | | | | |  |
| **To be completed in the morning** | | | | | | | | | |
| 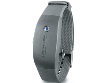**Time woke up:** | | | | | | | | |  |
| **Time got out of bed:** | | **Was proximity watch taken off?**  **Yes No** | | | | | | |  |
| **Estimated time taken to fall asleep:** | | | | | | | | |  |
| **To your knowledge, was the event marker pressed at the correct time? (Please circle)** | | | | | | | | |  |
| **Yes** | | | **No** | | | | | |  |
| **Please rate how typical your child’s sleep quality was**  **1 = Significantly better than usual, 3 = Typical and 5 = Significantly poorer than usual**  1 2 3 4 5 | | | | | | | | | |
| **Waking 1**  **Time of waking:**  **End of waking:**  **Perceived reason for waking (select one):**  Wet/needing toilet  Hungry/thirsty  Pain/discomfort  Anxiety  Unknown  Other ‑ please state:  **Waking 3**  **Time of waking:**  **End of waking:**  **Perceived reason for waking (select one):**  Wet/needing toilet  Hungry/thirsty  Pain/discomfort  Anxiety  Unknown  Other ‑ please state: | | | | | **Waking 2**  **Time of waking:**  **End of waking:**  **Perceived reason for waking (select one):**  Wet/needing toilet  Hungry/thirsty  Pain/discomfort  Anxiety  Unknown  Other ‑ please state:  **Waking 4**  **Time of waking:**  **End of waking:**  **Perceived reason for waking (select one):**  Wet/needing toilet  Hungry/thirsty  Pain/discomfort  Anxiety  Unknown  Other ‑ please state: | | | | |

Any other notes:

|  |
| --- |

Additional File 1: **Attachment** **S2:** Mobile sleep diary example


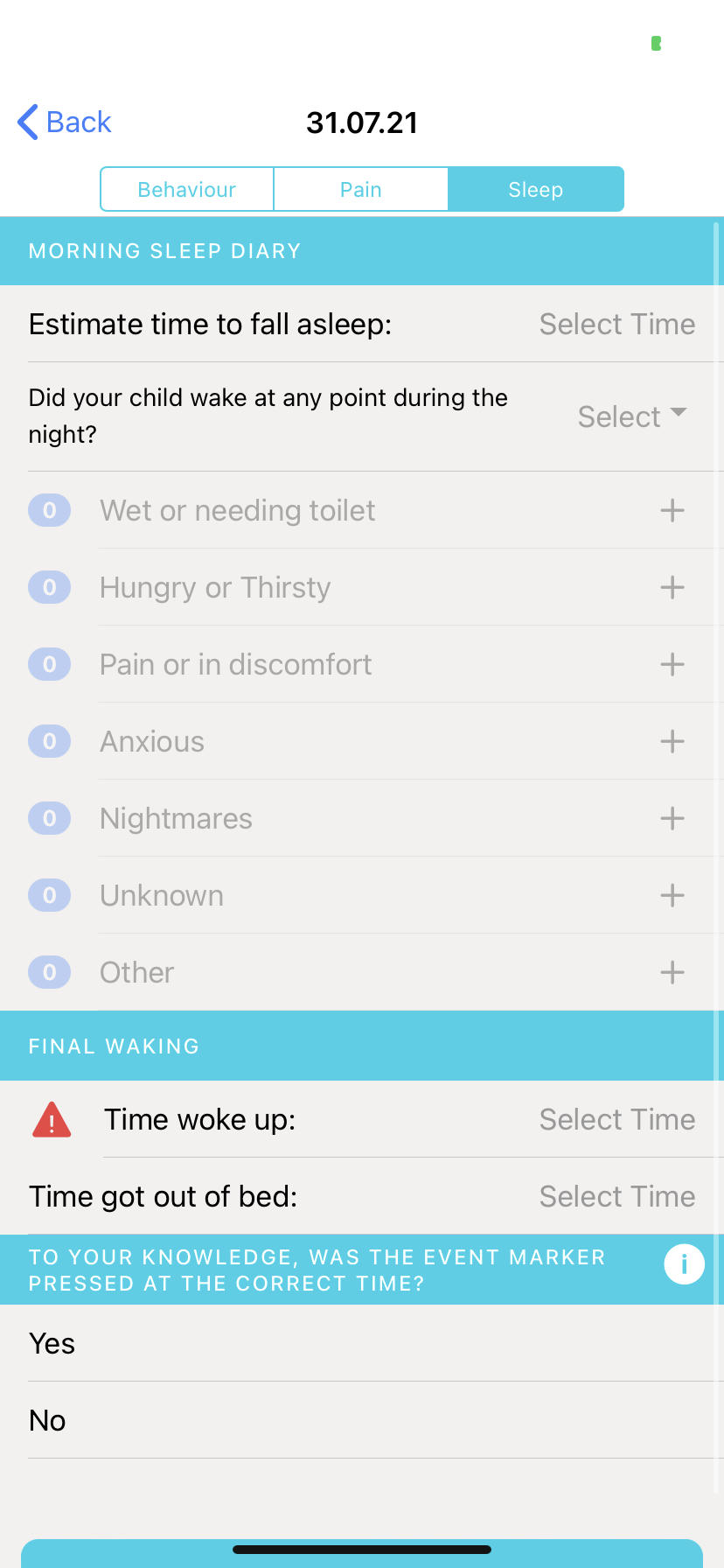

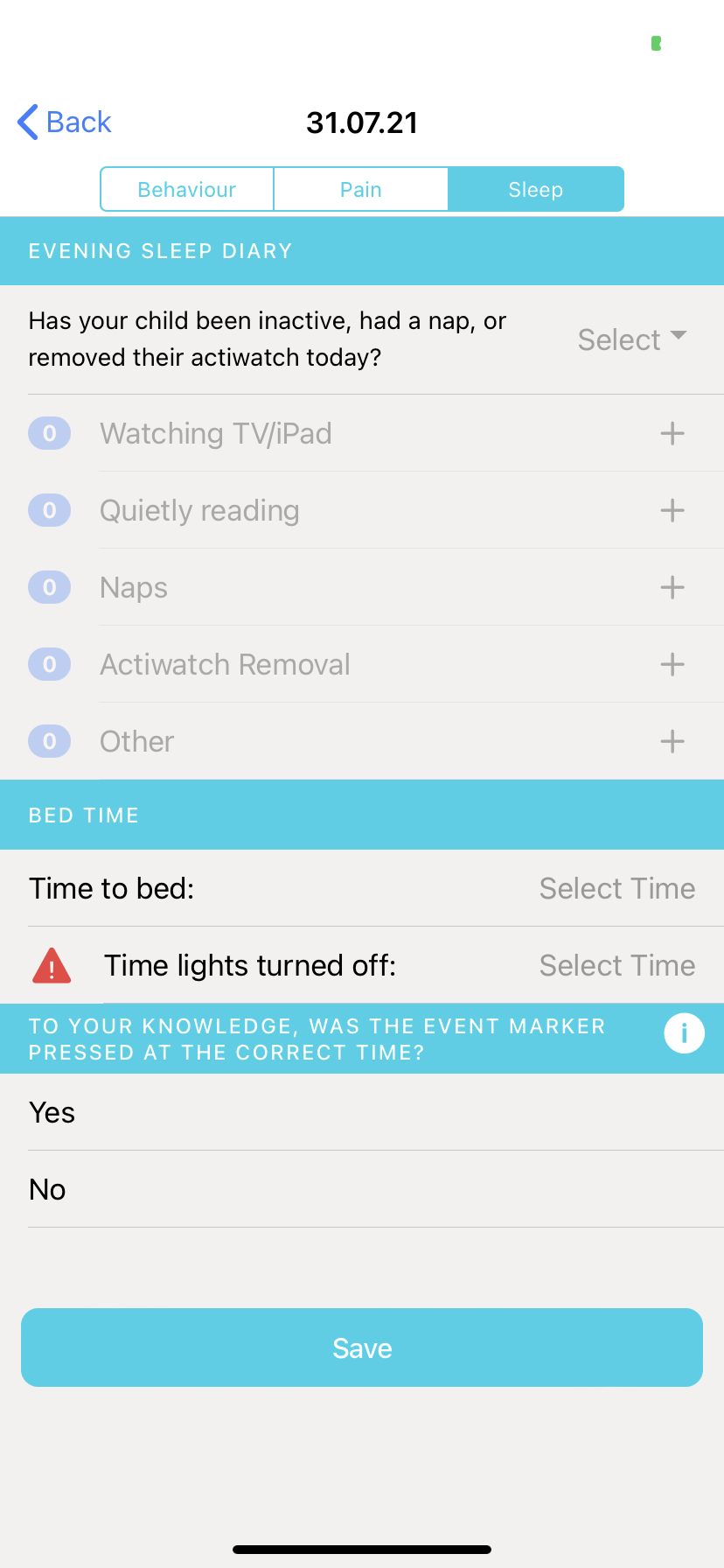

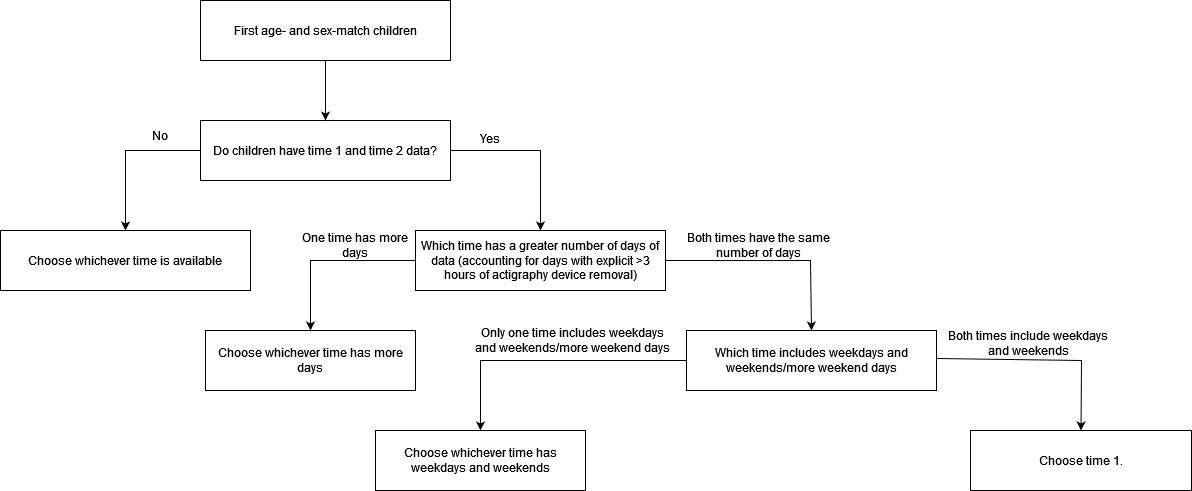


Additional File 1: **Figure S1:** Flow diagram for choosing the dataset to analyse

Additional File 1: **Attachment** **S3:** Actigraphy data cleaning protocol

Actigraphy cleaning protocol for overactivity analysis

Prerequisites before cleaning data:

- You have exported the actigraphy data into a .csv file, and opened this in Microsoft Excel (you need the part of the file with the data list showing the time of each epoch and the corresponding activity counts)
- You have the actogram for the actigraphy data open
- You have sleep diary data and actigraphy sleep intervals corresponding to the actigraphy data at-hand

1 – export the epoch-by-epoch list into Excel, reformat the columns and apply the formulae used to count 0 activity count epochs (referred to as ‘0 epochs’ throughout the remainder of the protocol). Ask ROS for formulae and template for reformatting the Excel document.

2 – visually check actogram. This should show when the sleep assessment began and finished. Choose midnight of the first complete 24-hour period of data collection as start point, end point is midnight after the last complete 24-hour period of data collection. Delete the rows in the Excel file containing data not within the chosen start and end times.

3 – check sleep diary data, it should indicate times when actiwatch has been removed on each day, as well as any periods of sedentary activity. If any times have been indicated, make a note of these times in the cleaning log.

4 – Check the epoch-by-epoch list for long periods of 0 epochs, long periods of 0 epochs indicate actiwatch removal. This allows you to find periods of actiwatch removal not reported in participant sleep diary. Note the times of the consecutive 0 epoch periods in the cleaning log.

- Different 0 epoch thresholds are used to indicate actiwatch removal during sleep and wake states (the sleep start and end times are indicated by the participant’s sleep intervals that should already be calculated). During sleep, two or more hours of consecutive 0 epochs indicate actiwatch removal (Trickett et al., 2017). During wake, 10 or more minutes of consecutive 0 epochs indicate actiwatch removal (Ekelund et al., 2004; Mattocks et al., 2008; Riddoch et al., 2004); this is unless the sleep diary states the child was napping/engaging in low-movement activity at the time.
- ROS has an Excel formula that automatically counts consecutive 0 epochs. If you have applied the consecutive 0 epoch-counting formula to the Excel file, just search for “20” and “240”. This will find 20 and 240 consecutive 0 epochs across the days, corresponding to 10 and 120 minutes of inactivity. You should be able to determine whether these periods of inactivity occur during wake or sleep, and whether periods of daytime inactivity overlap with sedentary periods recorded in the diary.

5 – add up the minutes from all periods of actiwatch removal, noted from the previous two steps, in each 24-hour period. Note the accumulated minutes in the cleaning log. Whilst doing this, follow the two principles below.

**Important notes for counting actiwatch removal time:**

1. Make sure not to double count any overlapping periods of removal indicated in diary and actogram (e.g. diary and actogram both indicate removal from 18:41:00-18:51:00 – total removal time is 10 mins, not 20 mins). If the diary and actogram periods partially overlap (e.g. diary removal = 18:41:00-18:50:30; actogram removal = 18:45:30-18:59:00), count all non-overlapping periods and do not double count overlapping periods.
2. Check the consecutive 0 epoch times against sedentary periods noted in the diary (this should be in the cleaning log).

Where there is no overlap between the consecutive 0 epochs and instances of low activity, count all the 0 epoch periods as removal time (see pyActigraphy tutorial doc).

If the 0 epoch period partially overlaps with instances of low activity in the diary, count the non-overlapping sections of the 0 epoch period as removal time where this **≥**10 consecutive minutes.

If the 0 epoch period completely overlaps with the low activity instance in the diary, then do not count this as removal time.

If removal time exceeds 3 hours (180 minutes) within a given 24-hour period, then the day should be excluded from the analyses (Rensen et al., 2020; Yavuz-Kodat et al., 2020). Make a note of any days that pass the 3 hour threshold in the cleaning log.

6 – If removal time does not exceed 3 hours within a given 24-hour period, then the removal times should be replaced with average activity counts from the corresponding times on the remaining included days (Shou et al., 2017; Tonetti et al., 2018; Yavuz-Kodat et al., 2020).

**References**

Ekelund, U., Yngve, A., Brage, S., Westerterp, K., & Sjöström, M. (2004). Body movement and physical activity energy expenditure in children and adolescents: how to adjust for differences in body size and age. *The American Journal of Clinical Nutrition, 79*(5), 851-856*.* <https://doi.org/10.1093/ajcn/79.5.851>

Mattocks, C., Ness, A., Deere, K., Tilling, K., Leary, S., Blair, S. N., & Riddoch, C. (2008). Early life determinants of physical activity in 11 to 12 year olds: cohort study. *British Medical Journal, 336*(7634), 26-29. <https://doi.org/10.1136/bmj.39385.443565.BE>

Rensen, N., Steur, L. M., Wijnen, N., van Someren, E. J., Kaspers, G. J., & van Litsenburg, R. R. (2020). Actigraphic estimates of sleep and the sleep-wake rhythm, and 6-sulfatoxymelatonin levels in healthy Dutch children. *Chronobiology International, 37*(5), 660-672. <https://doi.org/10.1080/07420528.2020.1727916>

Riddoch, C. J., Andersen, L. B., Wedderkopp, N., Harro, M., Klasson-Heggebø, L., Sardinha, L. B., ... & Ekelund, U. L. F. (2004). Physical activity levels and patterns of 9-and 15-yr-old European children. *Medicine & Science in Sports & Exercise, 36(*1), 86-92. <https://doi.org/10.1249/01.MSS.0000106174.43932.92>

Shou, H., Cui, L., Hickie, I., Lameira, D., Lamers, F., Zhang, J., ... & Merikangas, K. R. (2017). Dysregulation of objectively assessed 24-hour motor activity patterns as a potential marker for bipolar I disorder: results of a community-based family study. *Translational Psychiatry, 7*(8), e1211-e1211. <https://doi.org/10.1038/tp.2017.136>

Tonetti, L., Conca, A., Giupponi, G., Filardi, M., & Natale, V. (2018). Circadian activity rhythm in adult attention deficit hyperactivity disorder. *Journal of Psychiatric Research, 103*, 1-4. <https://doi.org/10.1016/j.jpsychires.2018.05.002>

Trickett J., Heald, M., Surtees, A., Clarkson, E., Agar, G., Oliver, C., & Richards, C. (2017). Actigraphy Cleaning Protocol. University of Birmingham.

Yavuz-Kodat, E., Reynaud, E., Geoffray, M. M., Limousin, N., Franco, P., Bonnet-Brilhault, F., ... & Schroder, C. M. (2020). Disturbances of continuous sleep and circadian rhythms account for behavioral difficulties in children with autism spectrum disorder. *Journal of Clinical Medicine, 9*(6), 1978*.* <https://doi.org/10.3390/jcm9061978>

| Additional File 1: **Table S1:** Median scores for M10 and M10 onset for each group, alongside between-group comparisons and post-hoc tests, following the exclusion of TD children recruited during COVID-19 national lockdowns. | | | | | | |
| --- | --- | --- | --- | --- | --- | --- |
| Table S1. Median scores for M10 and M10 onset for each group, alongside between-group comparisons and post-hoc tests, following the exclusion of TD children recruited during COVID-19 national lockdowns. | | | | | | |
|  | Group | | | | Between-group comparison | |
| Variable | AS | SMS | TSC | TD | *p* value | Post-hoc tests |
| Median M10  *(IQR)* | 283.47  *(173.46)* | 279.49  *(99.36)* | 258.35  *(129.07)* | 339.45  *(136.14)* | 0.063 | *p* = 0.21 – 1.00 |
| Median M10 onset  *(IQR)*^a^ | 08:52:00  *(98.50)* | 08:20:00  *(102.75)* | 09:01:30  *(117.25)* | 09:06:00  *(143.00)* | 0.076 | SMS earlier than TD (*p* = 0.075) |
| *Note.* AS: Angelman syndrome. IQR: interquartile range. SMS: Smith-Magenis syndrome. TD: typically-developing. TSC: tuberous sclerosis complex.  ^a^ Interquartile range presented in minutes. | | | | | | |

Additional File 1: **Figure S2:** Functional linear model and permutation F-test for 24-hour activity profiles of children with Angelman syndrome and typically-developing children, following exclusion of the typically-developing children recruited during COVID-19 national lockdowns.


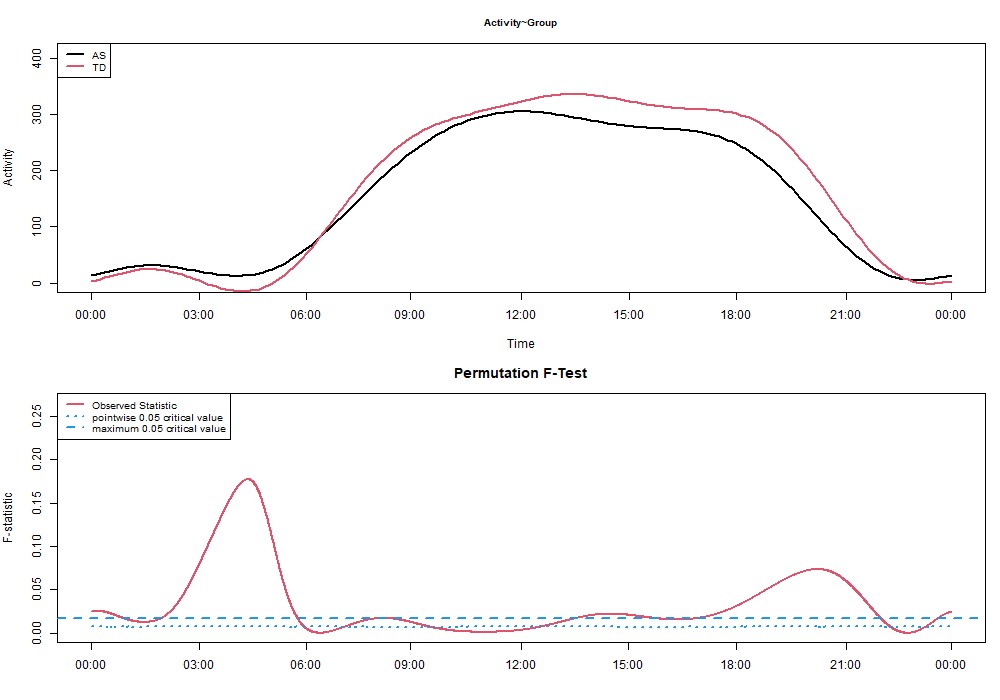


Additional File 1: **Figure S3:** Functional linear model and permutation F-test for 24-hour activity profiles of children with Smith-Magenis syndrome and typically-developing children, following exclusion of the typically-developing children recruited during COVID-19 national lockdowns.


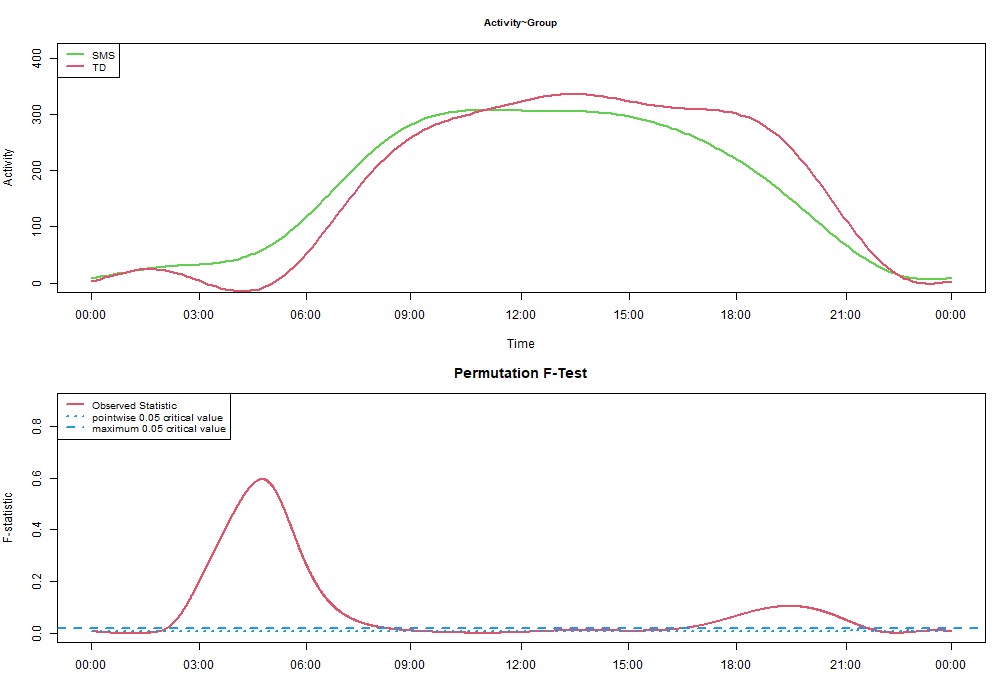


Additional File 1: **Figure S4:** Functional linear model and permutation F-test for 24-hour activity profiles of children with tuberous sclerosis complex and typically-developing children, following exclusion of the typically-developing children recruited during COVID-19 national lockdowns.


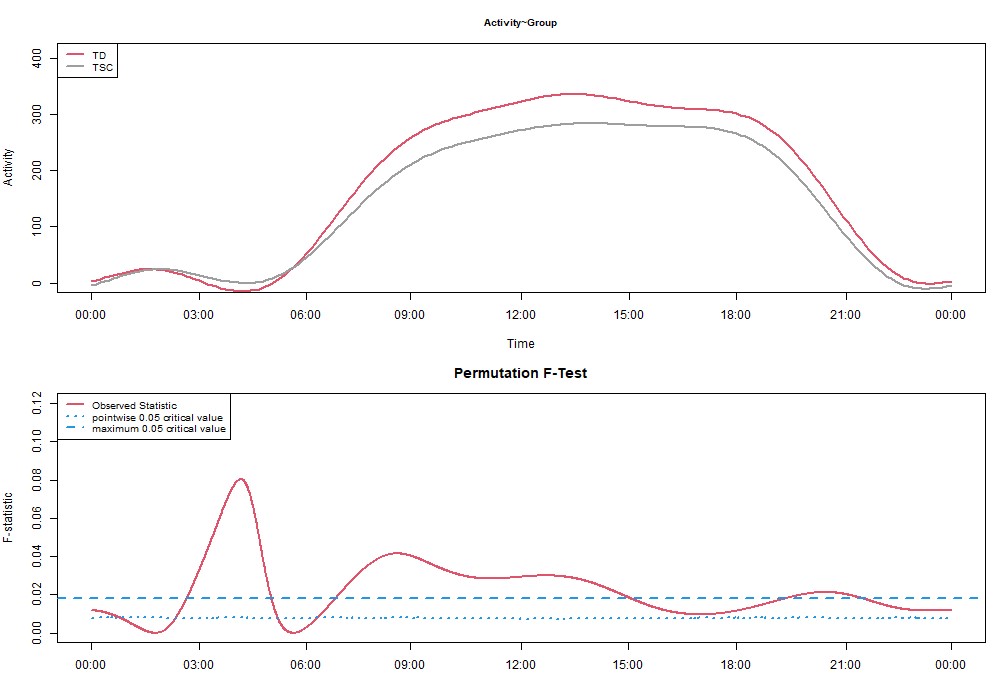


Additional File 1: **Table S2:** Correlations between M10 onset and TAQ overactivity subscale scores, across syndrome groups.

| Table S2. Correlations between M10 onset and TAQ overactivity subscale scores, across syndrome groups. | | |
| --- | --- | --- |
| Group | *r* value | *p* value |
| AS | -0.101 | 0.624 |
| SMS | -0.002 | 0.995 |
| TSC | 0.060 | 0.814 |
| *Abbreviations.* AS: Angelman syndrome. SMS: Smith-Magenis syndrome. TSC: tuberous sclerosis complex. | | |


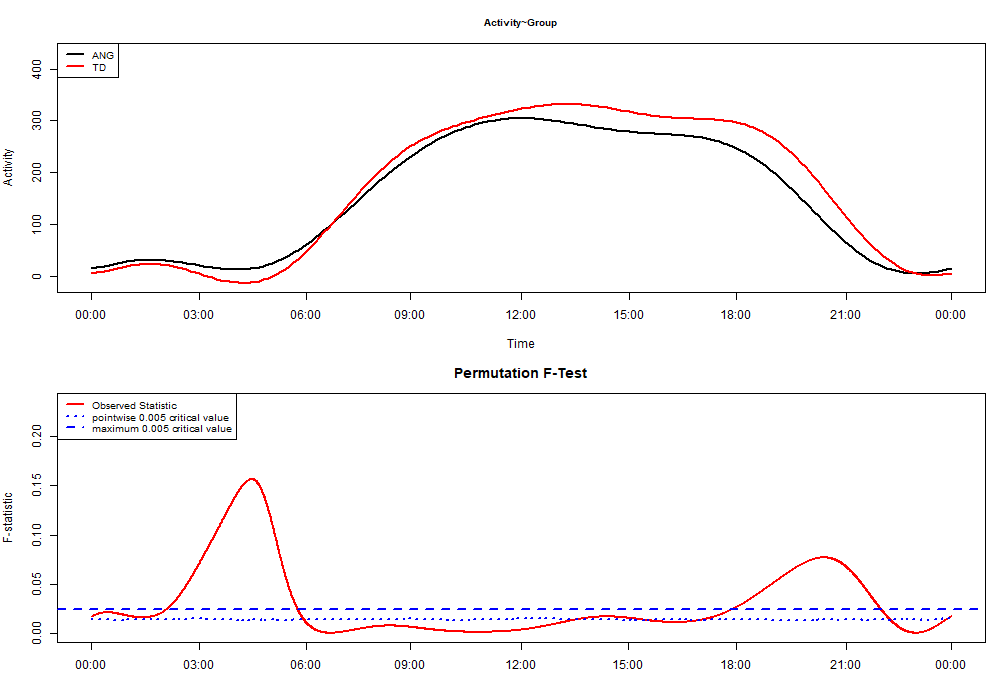


Additional File 1: **Figure S5:** FLM and permutation F-tests comparing 24-hour activity profiles between typically-developing and Angelman syndrome groups


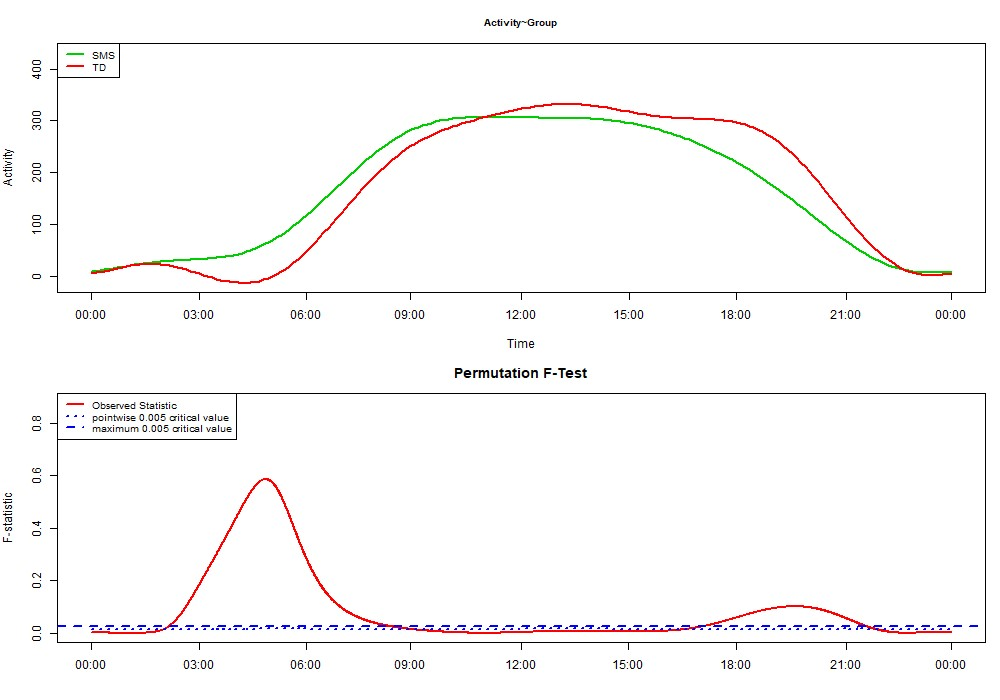


Additional File 1: **Figure S6:** FLM and permutation F-tests comparing 24-hour activity profiles between typically-developing and Smith-Magenis syndrome groups


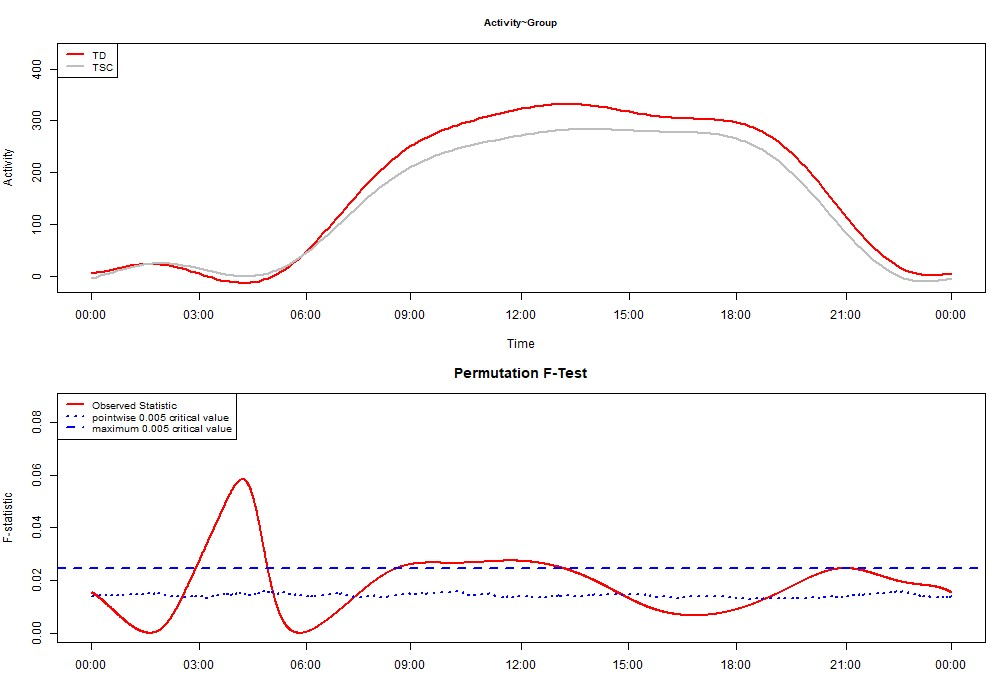


Additional File 1: **Figure S7:** FLM and permutation F-tests comparing 24-hour activity profiles between typically-developing and tuberous sclerosis complex groups
